# Progress in Diseases of the Heart

**Published:** 1900-10-27

**Authors:** 


### Progress in Diseases of the Heart.

(*Continued from page* 52.)

**Rupture of the Heart.**—Goodman[26] records a case of rupture of the heart with coincident hæmorrhage into the pons varolii. The patient had had rheumatic fever several years previously, and under the influences of emotion and brandy became comatose suddenly and died.

**Aneurysm.**—Hall[27] states that in the differentiation between malignant growth and aneurysm it is important to recognise the difference between tracheal tug and tracheal pulsation. He quotes Toulmin, that the pulsation is seen in other diseases and is affected by respiration, often being only present during inspiration. To aid the diagnosis Hall describes "the transmission of the diastolic shock originating at the time of closure of the aortic valves, through the aneurysm to the trachea, and manifested by a distinct sharp impulse following the tracheal tug at the same interval as that between the apex beat and the closure of the aortic segments." The diastolic shock has been acknowledged of great importance when the aneurysmal tumour has reached the chest wall, but this being noticeable earlier is at least of equal importance. Hale White[28] says "whenever, in the chest, you have any sign for which you cannot find a definite cause, and which persists, then it may be due to aneurysm. Again, if bronchitis occurs without apparent cause, and will not clear up under treatment, it is well to think of aneurysm. Dysphagia and paralysis of the left vocal cord, brassy cough, deficient entry of air on right side, are all symptoms that may guide one to aneurysm. More aneurysms are overlooked than should be, since people wait for so many symptoms." Heller[29] points out, after observing a large number of cases of aneurysm, that the cases due to syphilis exceed considerably those due to other causes. Burt[30] records a case of aneurysm of descending arch of aorta rupturing into the trachea. Cabot[31] gives notes of a case of thoracic aneurysm which perforated the sternum in two places without causing pain or pressure symptoms. Bishop[32] extols the use of iodides in treatment of aneurysm, and Conner reports three cases of the treatment of aneurysm by injection of gelatine. He uses 2-1 per cent., and in one of the three cases improvement seemed to follow its use, but there was always great local pain at the site of the injections.

Lediard[33] has successfully removed a subclavian aneurysm after ligature of the first part of the axillary had proved a failure. The arm, which previously had grown useless, now at once regained its functions. Buchholz[34] reports the case of an aneurysm, the size of the fist, situated in the epigastric part of the aorta, producing vomiting, stomach-pain, tympanites and insomnia. A 10 per cent. solution of gelatine, in normal salt solution, was given by the mouth, and the patient kept recumbent with an ice-bag night and day. Drugs were also given for the general condition. After two months the gelatine was given every second day, and the ice-bag worn two hours daily. A month later the patient was able to return to work, the aneurysm being cured. This the writer considers due to the combined effect of the ice and the gelatine.

**Influenza and Heart Disease.**—Saundby[35] holds that the cardiac troubles of influenza may be functional or organic. Of the former bradycardia, as part of a general enfeeblement, is most common, or tachycardia, with irregularity and intermission. These conditions may be variable, and may only be present under special circumstances, e.g. fatigue, and are present chiefly in females. Of the organic, dilatation, often extreme, is commonest, with all its attendant troubles. Functional may pass into organic trouble, and there may be subacute myocarditis and fatty degeneration. In functional disorders he recommends rest, care over the general health: iron and arsenic, digitalis may be useful in tachycardia. For dilatation he recommends the Nauheim treatment.

**Dilatation.**—Stacey Wilson[36] divides right ventricular dilatation into three classes—(1) those where pulsation appears in vessels of neck; (2) those where the pulsation appears downwards to liver; (3) those where there is little if any pulsation, but the dilatation affects mostly the conus arteriosus.

**Complications of Heart Disease.**—*Mitral Dwarfing.*—Gilbert and Rathcry[37] describe two patients with "mitral dwarfing." They presented "diminution in volume of all parts and an exceedingly small stature." Numerous irregularities or absence of development are common in such cases, and mental development may be very backward. The infantile type may dominate the genital organs, with accompanying sterility.

*Venous Thrombosis.*—Welch[38] records four and has collected 23 cases of venous thrombosis in cardiac disease. This he considers to be due probably to micro-organisms, though only in one case was a micro-organism found. Seventeen cases were in women, four in males, two not stated. The thrombus appeared in the arm and neck commonly, frequently in these two situations together Age, 15-30. It occurred usually during failure of compensation.

*In Mitral Stenosis.*—Huchard[39] urges the importance of a careful consideration as to the etiology of mitral stenosis. He quotes a case, as also does Gairdner,[40] illustrating that pregnancy in mitral stenosis is not necessarily so dangerous a complication as formerly supposed. Huchard recommends absolute rest and quiet after the fourth month. He considers pure mitral stenosis is congenital and of a hereditary, syphilitic nature. Frequently other congenital malformations are found coincidently with the stenosis, e.g., absence of palate, harelip and patent foramen ovale. The auscultatory signs are very slight sometimes, and hemiplegia may be coincident, without the stenotic condition being discovered.

**Diphtheritic Heart.**—Rolly[41] sums up his investigations on the effect of diphtheritic toxin on the heart thus: Paralysis of the heart follows immediately the fall of blood pressure caused by the paralysis of the vaso-motor centre, and leads to death in spite of artificial respiration. Poynton[42] compares the degeneration of the muscle fibres in diphtheria with that occurring in acute rheumatism and chorea. In diphtheria the heart-muscle degenerates as a result of the toxins, and dilatation is the result of this degeneration. In acute rheumatism dilatation occurring apart from valvular or pericardial inflammation may be due to the action of toxins. He shows that the degeneration of cardiac muscle in rheumatism and chorea is very similar to that in diphtheria. This suggestion does not necessarily imply that the toxin is of bacterial origin, but, short of absolute proof, the infectious origin of rheumatic fever seems to rest upon very strong evidence.

[26] Lancet, April 14. [27] Amer. Jour. Med. Sci., Jan. [28] Clin Jour., Mar. 28. [29] Brit. Med. Jour., June 9. [30] Med. Rec., April 28. [31] Amer. Jour. Med. Sci., April. [32] Med. Rec., May 12. [33] Brit. Med. Jour., May 5. [34] Brit. Med. Jour., Mar. 10. [35] Practitioner, May. [36] Birm. Med. Rev., June. [37] New York Med. Jour., June 2. [38] Brit. Med. Jour., June 2, [39] Brit. Med. Jour., Mar. 24. [40] Clin. Jour., June 27. [41] Post Graduate. May. [42] Lancet, May 12.

